# Length of hospitalization in intensive care units: A cross-sectional study

**DOI:** 10.1097/MD.0000000000049222

**Published:** 2026-06-12

**Authors:** Reema Karasneh, Sayer Al-Azzam, Karem H. Alzoubi, Lana Rababah, Dua’a Mohammad, Dania Rahhal, Louai Alsaloumi, Suad Kabbaha, Mamoon A. Aldeyab, Ohoud Aljuhani

**Affiliations:** a Department of Basic Pathological Sciences, Faculty of Medicine, Yarmouk University, Irbid, Jordan; b Department of Clinical Pharmacy, Jordan University of Science and Technology, Irbid, Jordan; c Department of Pharmaceutical Sciences, College of Pharmacy, Qatar University, Doha, Qatar; d Department of Pharmacy Practice and Pharmacotherapeutics, College of Pharmacy, University of Sharjah, Sharjah, UAE; e Faculty of Pharmacy, Jordan University of Science and Technology, Irbid, Jordan; f Department of Health, University of Essex, Colchester, UK; g Department of Applied Pharmaceutical Sciences and Clinical Pharmacy, Faculty of Pharmacy, Isra University, Amman, Jordan; h Department of Health Research Methods, Evidence & Impact (HEI), McMaster University, Hamilton, ON, Canada; i School of Pharmacy, University of Reading, Whiteknights, Reading, UK; j Department of Pharmacy Practice, Faculty of Pharmacy, King Abdulaziz University, Jeddah, Saudi Arabia; k Saudi Society for Multidisciplinary Research Development and Education (SCAPE Society), Saudi Arabia.

**Keywords:** comorbidities, critical care, intensive care units, length of stay, predictors

## Abstract

Prolonged hospitalizations in intensive care units (ICUs) due to critical conditions are a significant challenge to the healthcare system. The primary objective within the ICU is to reduce the length of stay (LOS), which ultimately could enhance the quality of healthcare, minimize complications, and reduce healthcare-associated costs. This study aims to identify factors affecting the LOS in ICUs. This is a retrospective cross-sectional study that utilized electronic records from a tertiary hospital, analyzing data from 7628 ICU admissions between 2012 and 2022. A total of 7628 ICU patients were included, with a mean stay of 4.3 ± 7.3 days, 33.7% remained ≥ 3 days. The majority of the included patients were males (59.12%) and aged above 60 years (53.03%). Several factors were significantly associated with prolonged ICU stay including sepsis (odds ratio [OR] = 5.56, 95% confidence interval [CI]: 3.42–9.04, *P* < .001), receiving nutrition via a nasogastric (NG) tube (OR = 2.42, 95% CI: 1.88–3.13, *P < *.001), and receiving antibiotics (OR = 2.07, 95%CI: 1.81–2.38, *P* < .001). In generalized linear model analysis, tracheostomy, sepsis and mechanical ventilation were strongest predictors of prolonged ICU stay (Exp(B) = 3.33, 1.63, 1.60), respectively, *P* < .001). This study identified several factors significantly associated with LOS in the ICU. Illness severity, intensity of supportive care, and patterns of clinical management were the main contributors to prolonged ICU stay. Identifying these factors can help optimize patient care, minimize complications, and alleviate healthcare costs within critical care settings, thereby informing the efficient allocation of resources.

**Key messages**

- Antibiotic use, supportive care (e.g., tracheostomy, enteral feeding support, mechanical ventilation), and greater illness severity (e.g., sepsis/septic shock, DIC, vasopressor-dependent hypotension, stroke) significantly impact ICU length of stay.
- Identifying key determinants of LOS can improve patient management for optimal outcomes.
- Findings of the study can help in developing strategies that reduce resource utilization in the ICU.

## 1. Introduction

Length of Stay (LOS) is a critical issue within hospitals and healthcare facilities worldwide. Prolonged hospital stays can lead to hospital-acquired complications such as healthcare-associated infections, falls, and thrombosis, causing increased stress and healthcare costs for both patients and hospitals.^[1]^ Intensive care units (ICUs) are special care settings developed for critically ill patients to perform close monitoring, rapid intervention, continuous follow-up, and treatment of acute disease.^[2]^ Prolonged ICU LOS does not only increase direct medical costs but also contributes to indirect costs such as delayed bed turnover or extended hospital stays. Prior studies have estimated that each additional ICU day can significantly increase hospitalization costs, especially in low- and middle-income countries.^[3–5]^ Timely discharge from hospitals and reducing the LOS can mitigate direct and indirect costs and improve patient satisfaction.

Several factors were identified as affecting LOS in the ICU, including patient-related factors, baseline health conditions, laboratory parameters, and certain medications.^[6]^ Advanced age is one of the most important variables associated with prolonged ICU stays due to the need for prolonged monitoring and specialized care.^[7–9]^ Similarly, cardiovascular and renal diseases were consistently associated with more extended stays as they require prolonged monitoring and specialized care.^[10]^ This aligns with the results of a retrospective study conducted in 2018 on 3925 patients, which found a significant increase in LOS in patients with cardiovascular diseases, multiple diseases, nervous system diseases, and cerebrovascular diseases (*P *< .05).^[11]^ Moreover, the same study showed that when urea, creatinine, and sodium values increase in parallel, the LOS increases (*P < *.05).^[11]^ However, other factors remain uncertain, including gender and medication use.^[12,13]^

Understanding these determinants noticeably enhances patient care and resource allocation in ICU settings. Several studies have found that prolonged stays distress patients and families and escalate healthcare costs and resource utilization.^[14,15]^ A recent study conducted in Jordan in 2022 assessed hospital unit costs and reported that ICU specialties presented the highest unit costs per patient day across providers (JOD 377.800 = USD 532.90), and the patient-day cost was JOD 236.600 (USD 333.20), emphasizing the need for LOS reduction.^[16]^ On the other hand, longer LOS in the ICU is associated with increased mortality.^[17]^ Therefore, the primary objective is to reduce LOS in the ICU and thus health care expenses.^[11]^

The average length of stay (ALOS) in hospitals is defined as the mean number of days spent in a hospital and varies among different countries and populations. According to the Organization for Economic Cooperation and Development (OECD) in 2022 ALOS was the longest in Japan (16 days) and Portugal (9.1 days), while in the United States, it was 5.9 days.^[18]^ In Saudi Arabia, the overall median LOS in the ICU was 3 days.^[6]^ Limited reports were found on ALOS in Jordan that included a study conducted by Hammad et.al and indicated that the ALOS was 3.7 days in 2022.^[16]^ Therefore, examining factors that may affect LOS in the ICU may help enhance patient outcomes and initiate efficient treatment plans to reduce LOS. To our knowledge, limited studies investigate factors affecting LOS in the ICU in Jordan. Thus, this study aimed to determine the average length of ICU stay (ALOS) among ICU patients and identify demographic and other factors that affect LOS in the ICU in Jordan.

## 2. Materials and methods

### 2.1. Participants

This single-center retrospective study was conducted using electronic health records at a tertiary hospital. The study included all records for patients who were admitted to the ICU from July 2012 to July 2022. The sample consisted of 26,248 ICU admissions for 13,567 patients and only the first ICU admission per patient was included. Patients aged < 18 years, diagnosed with cancer or those who have missing data for LOS were excluded.

### 2.2. Procedure

Using electronic medical records, data on demographic variables (e.g., age, gender, smoking status), medical history and comorbidities, prescription medications, ICU procedures, complications and the first laboratory readings at ICU admission were obtained. Dates of ICU admission and discharge were also extracted.

### 2.3. Data analysis

Statistical analyses included descriptive statistics with categorical variables summarized as frequencies and percentages, and continuous variables presented as means and standard deviations (SD). The Kolmogorov-Smirnov test was used to assess the normality of data distribution. An independent t-test was conducted to compare means where appropriate and the Pearson Chi-square test was used to assess associations between categorical variables.

Logistic regression analysis was performed to identify factors associated with ICU stays of 3 days or longer. In addition, a generalized linear model (GLM) with gamma distribution and log link was applied to further explore predictors of ICU length of stay (LOS) as a continuous outcome. Independent variables were selected for both models using a backward stepwise approach, retaining variables with *P* < .25. A significance level of α = 0.05 was adopted. All statistical analyses were conducted using IBM SPSS Statistics, Version 25.0.

### 2.4. Ethical consideration

The Institutional Review Board of Yarmouk University (Reference no IRB/2024/067) granted ethical approval. Informed consent was waived by the IRB as the study uses anonymized electronic health records.

## 3. Results

After screening and applying eligibility criteria, 7628 patients who were hospitalized in the ICU from July 2012 to July 2022 met the criteria and were included in the final analysis. Of these, 4510 (59.12%) were male, and more than half were 60 years of age or older (53.03%) and nonsmokers (55.32%). The mean length of stay (LOS) in the ICU was 4.296 ± 7.307 days, with 66.3% of participants hospitalized for less than 3 days. LOS was significantly associated with both age and smoking (*P* < .001) (Table 1).

**Table 1:** Characteristics of the admitted ICU patients.

Variables	Overall (n = 7628)	Length of stay (mean: 4.296 ±* 7.307)		Pearson chi-square	*P* value
	N (%)	<3 days N (%)	≥ 3 days N (%)		
		5056 (66.3%)	2572 (33.7)		
Age					
18–29	790 (10.36%)	547 (69.24%)	243 (30.76%)	40.376	**<.001**
30–39	722 (9.47%)	496 (68.7%)	226 (31.3%)		
40–49	868 (11.38%)	590 (67.97%)	278 (32.03%)		
50–59	1203 (15.77%)	848 (70.49%)	355 (29.51%)		
60–69	1387 (18.18%)	933 (67.27%)	464 (33.45%)		
70 or above	2658 (34.85%)	1642 (61.78%)	1016 (38.22%)		
Gender					
Male	4510 (59.12%)	3015 (66.85%)	1495 (33.15%)	0.28	.614
Female	3118 (40.88%)	2041 (65.46%)	1077 (34.54%)		
Smoking state					
Smoker	1639 (21.49%)	1230 (75.05%)	409 (24.95%)	78.303	**<.001**
Never smoking	4220 (55.32%)	2655 (62.91%)	1567 (37.13%)		
Ex smoker	420 (5.51%)	285 (67.86%)	135 (32.14%)		

Data are expressed as mean and standard deviation for continuous variables and Frequency (%) for categorical variables.

* ±: Standard deviation.

As shown in Table 2, HTN was the most prevalent health condition among ICU patients, accounting for 44.81%, followed by DM (36.59%). On average, patients had nearly 2 comorbid diseases (mean ± SD = 1.87 ± 1.568). Approximately three-quarters of patients with IHD (72.98%), hyperlipidemia (74.21%), or asthma (72.3%) stayed in the ICU for less than three days. Regarding medication use, 2005 (26.28%) patients were receiving beta-blockers, and 4682 (61.38%) were on antibiotics with higher proportions observed among patients with ICU stays of < 3 days compared with those with longer stays (*P* < .001). The LOS was significantly associated with stroke (*P* < .001), IHD (*P* < .001), hyperlipidemia (*P* = .011), and DVT (*P* = .04) with affected patients demonstrated shorter ICU stays compared with those without these conditions.

**Table 2:** Chronic diseases and medications used by ICU-admitted patients.

Variables	N (%)	Length of stay		Pearson chi-square	*P* value
		<3 days N (%)	≥ 3 days N (%)		
		5056 (66.3%)	2572 (33.7)		
Hypertension (HTN)	3418 (44.81%)	2258 (66.06%)	1160 (33.94%)	0.134	.714
Diabetes (DM)	2791 (36.59%)	1852 (66.36%)	939 (33.64%)	0.011	.917
Strok	792 (10.38%)	435 (54.92%)	357 (45.08%)	51.012	**<.001**
Ischemic heart disease (IHD)	1251 (16.4%)	913 (72.98%)	338 (27.02%)	30.052	**<.001**
Atrial fibrillation (AF)	423 (5.55%)	272 (64.3%)	151 (35.7%)	0.785	.376
Hyperlipidemia	221 (2.9%)	164 (74.21%)	57 (25.79%)	6.398	**.011**
Heart failure (HF)	642 (8.42%)	440 (68.54%)	202 (31.46%)	1.593	.207
Chronic kidney disease (CKD)	496 (6.5%)	329 (66.33%)	167 (33.67%)	0.001	.981
ESRD	271 (3.55%)	183 (67.53%)	88 (32.47%)	0.195	.659
COPD	158 (2.07%)	110 (69.62%)	48 (30.38%)	0.804	.37
Asthma	148 (1.94%)	107 (72.3%)	41 (27.7%)	2.443	.118
Deep vein thrombosis (DVT)	120 (1.57%)	69 (57.5%)	51 (42.5%)	4.207	**.04**
Gout	158 (2.07%)	110 (69.62%)	48 (30.38%)	0.804	.37
Number of comorbidities*	1.87 ± 1.568	1.86 ± 1.578	1.89 ± 1.55	_	.426
Beta blockers	2005 (26.28%)	1223 (61%)	782 (39%)	33.988	**<.001**
Antibiotics	4682 (61.38%)	2647 (56.54%)	2035 (43.46%)	515.283	**<.001**

COPD = chronic obstructive pulmonary disease, ESRD = end stage renal disease.

Table 3 presents the association between LOS and laboratory parameters among the admitted ICU patients. Those who stayed in the ICU for 3 days or longer had significantly higher levels of creatinine, urea, sodium, alkaline phosphatase, white blood cell (WBC) count, HbA1c, and PO_2_, and significantly lower levels of potassium, albumin, calcium, and hemoglobin compared to those who stayed less than 3 days.

**Table 3:** Laboratory results for ICU admitted patients.

Variables	Overall	Length of stay		Mean Difference	t (df)	*P* value	95% CI
	Average	<3 days	≥ 3 days				
Creatinine umol/l	149.37 ± 175.64	142.49 ± 172.18	161.37 ± 180.92	−18.882	−4.32 (6951)	<.001	(−27.45, −10.314)
Urea mmol/l	11 ± 10.1	10 ± 9.26	12.72 ± 11.21	−2.725	−10.859 (6859)	<.001	(−3.217, −2.233)
Potassium mmol/l	4.31 ± 0.96	4.34 ± 1.05	4.25 ± 0.8	0.089	3.695 (6953)	<.001	(0.042, 0.136)
Sodium mmol/l	139.81 ± 6.87	138.84 ± 5.95	141.49 ± 7.96	−2.645	−15.711 (6948)	<.001	(−2.974, −2.315)
Aspartate Aminotransferase UL	92.68 ± 413.23	97.6 ± 464.21	85.15 ± 319.77	12.44	1.052 (5108)	.293	(−10.741, 35.621)
Alanine Transaminase UL	60.71 ± 244.05	64.55 ± 280.67	54.83 ± 173.47	9.724	1.39 (5092)	.164	(−3.987, 23.435)
Alkaline Phosphatase	156.76 ± 181.39	151.23 ± 178.43	165.2 ± 185.54	−13.977	−2.675 (5032)	.007	(−24.219, −3.735)
Albumin g/l	33.3 ± 7.55	34.88 ± 7.36	30.9 ± 7.21	3.986	18.884 (4990)	<.001	(3.572, 4.399)
Calcium mmol/L	2.12 ± 0.23	2.15 ± 0.24	2.07 ± 0.23	0.077	12.055 (5535)	<0.001	(0.064, 0.089)
Magnesium mmol/L	0.82 ± 0.17	0.82 ± 0.17	0.82 ± 0.17	−0.005	−0.983 (5536)	.326	(−0.014, 0.005)
Phosphorus mmol/L	1.26 ± 0.57	1.27 ± 0.56	1.25 ± 0.59	0.024	1.536 (5321)	.125	(−0.007, 0.056)
Hemoglobin g/dl	11.14 ± 2.43	11.42 ± 2.47	10.67 ± 2.29	0.745	12.395 (6868)	<.001	(0.627, 0.863)
White Cell Count 10*3/mm*3	12.29 ± 9.43	12.07 ± 10.3	12.67 ± 7.71	−0.594	−2.521 (6865)	.012	(−1.056, −0.132)
Platelets 10*3/mm*3	239.79 ± 118.46	241.87 ± 112.65	236.23 ± 127.68	5.642	1.906 (6865)	.057	(−0.161, 11.445)
Glucose mol/l	10.62 ± 6.99	10.61 ± 6.95	10.65 ± 7.08	−0.038	−0.126 (2459)	.9	(−0.624, 0.548)
HbA1C (Glycated Hemoglobin) %	8.01 ± 8.25	7.65 ± 6.19	8.69 ± 11.16	−1.04	−2.845 (2252)	.004	(−1.757, −0.323)
HCO3 mmol/l	23.01 ± 25.06	22.56 ± 32.63	23.56 ± 9.73	−0.995	−1.344 (4629)	.179	(−2.446, 0.456)
PCO2 mm Hg	40.45 ± 55.03	40.77 ± 72.34	40.06 ± 18.35	0.708	0.436 (4628)	.663	(−2.479, 3.896)
PO2 mm hg	82.59 ± 51.09	78.48 ± 51.48	87.59 ± 50.18	−9.104	−6.051 (4620)	<.001	(−12.054, −6.154)

As presented in Table 4, 86.38% of patients were admitted to the ICU due to medical conditions. More than two-thirds of patients who were on mechanical ventilation (69.08%), underwent tracheostomy (89.03%), or received nutrition via a nasogastric (NG) tube (70.72%) or percutaneous endoscopic gastrostomy (PEG) tube (77.03%) remained in the ICU for 3 days or longer (*P* < .001). Similarly, patients who developed complications stayed in the ICU for ≥ 3 days compared to those who did not (*P* < .001).

**Table 4:** ICU procedures and complications according to ICU length of stay.

Variables	Overall (n = 7628)	Length of stay		Pearson chi-square	*P* value
		(mean: 4.296 ±* 7.307)			
	N (%)	<3 days	≥ 3 days		
Type of Admission					
Medical	6589 (86.38%)	4317 (65.52%)	2272 (34.48%)	11.363	.001
Surgical	985 (12.91%)	699 (70.96%)	286 (29.04%)		
Mechanical Ventilation MV (invasive)	1475 (19.34%)	456 (30.92%)	1019 (69.08%)	1035.687	<.001
MV at admission (including CPAP)	525 (6.88%)	203 (38.67%)	322 (61.33%)	187.463	<.001
Tracheostomy	155 (2.03%)	17 (10.97%)	138 (89.03%)	216.366	<.001
Renal Replacement Therapy (RRT)	379 (4.97%)	170 (44.85%)	209 (55.15%)	83.323	<.001
NG Tube	1284 (16.83%)	376 (29.28%)	908 (70.72%)	990.943	<.001
PEG Tube	148 (1.94%)	34 (22.97%)	114 (77.03%)	126.057	<.001
Complications					
Sepsis/ septic shock	404 (5.3%)	77 (19.06%)	327 (80.94%)	478.285	<.001
DIC	204 (2.67%)	59 (28.92%)	145 (71.08%)	149.29	<.001
Hypotension on vasopressors	755 (9.9%)	251 (33.25%)	504 (66.75%)	479.604	<.001
None	5161 (67.66%)	4001 (77.52%)	1161 (22.5%)	1031	<.001
Vasopressor use at admission	164 (2.15%)	91 (55.49%)	73 (44.51%)	8.346	.004
CPR	2040 (26.74%)	991 (48.58%)	1049 (51.42%)	382.997	<.001

CI = confidence interval, CPAP = continuous positive airway pressure, CPR = cardiopulmonary resuscitation, DIC = disseminated intravascular coagulation, NG = nasogastric, PEG = percutaneous endoscopic gastrostomy.

Determinants of intensive care unit stay lasting 3 days or longer. are shown in Table 5. Invasive mechanical ventilation, vasopressor support, renal replacement therapy, sepsis/septic shock, disseminated intravascular coagulation (DIC), and tracheostomy were found to be significantly associated with longer ICU length of stay (*P* < .001). Additionally, patients who received antibiotics had 2.07 times higher odds of staying in the ICU for 3 days or longer compared with those who did not receive antibiotics (odds ratio [OR] = 2.07; 95% confidence interval [CI] 1.81–2.38, *P *< .001). On the other hand, smoking was significantly associated with 40% lower odds of prolonged LOS (OR = 0.60; 95% CI: 0.51–0.70, *P *< .001) compared to nonsmokers (Table 5).

**Table 5:** Determinants of intensive care unit stay lasting 3 days or longer.

	Odds ratio	95% CI		*P* value
Antibiotics	2.07	1.81	2.38	<.001
Smoking status				
Never Smoking				
Smoker	0.60	0.51	0.70	<.001
Ex Smoker	0.76	0.58	1.00	.051
Mechanical Ventilation MV (invasive)	2.92	2.19	3.89	<.001
NG Tube	2.42	1.88	3.13	<.001
PEG Tube	2.52	1.23	5.14	.011
Sepsis/ septic shock	5.56	3.42	9.04	<.001
Hypotension on vasopressors	2.38	1.79	3.16	<.001
DIC	2.64	1.61	4.32	<.001
Vasopressor use at admission	0.88	0.50	1.53	.240
Stroke	1.71	1.39	2.11	<.001
Tracheostomy	2.87	1.26	6.54	<.001
Type of admission				
Medical				
Surgical	0.87	0.72	1.05	.151
Renal Replacement Therapy (RRT)	1.71	1.24	2.35	.001
CPR	0.74	0.59	0.92	.006

CI = confidence interval, CPR = cardiopulmonary resuscitation, DIC = disseminated intravascular coagulation, MV = mechanical ventilation, NG = nasogastric, PEG = percutaneous endoscopic gastrostomy.

In the GLM with a gamma distribution and log link shown in Table 6, the use of antibiotics was associated with a 46% increase in the expected ICU length of stay (Exp(β) = 1.46, *P* < .001), compared with patients who did not receive antibiotics. On the other hand, surgical ICU admission was associated with a 10% shorter expected ICU stay compared with medical ICU admission (Exp(β) = 0.90, *P* = .009). Further details of the GLM analysis, are shown in Table 6.

**Table 6:** Predictors of ICU length of stay (generalized linear model analysis).

b	Coefficient	Exp (coefficient)	95%CI		*P* value
Age					
18–29	Ref				
30–39	0.064	1.07	0.96	1.19	.246
40–49	0.071	1.07	0.97	1.19	.173
50–59	−0.028	0.97	0.88	1.07	.225
60–69	−0.048	0.95	0.87	1.05	.333
70 or above	−0.054	0.95	0.87	1.04	.248
Antibiotics	0.381	1.46	1.39	1.55	<.001
Mechanical Ventilation MV (invasive)	0.468	1.60	1.45	1.76	<.001
Tracheostomy	1.202	3.33	2.75	4.03	<.001
NG Tube	0.375	1.46	1.33	1.60	<.001
PEG Tube	0.262	1.30	1.07	1.57	.007
Sepsis/ septic shock	0.491	1.63	1.45	1.84	<.001
DIC	0.393	1.48	1.27	1.73	<.001
Hypotension on vasopressors	0.178	1.19	1.09	1.31	<.001
CPR	0.039	1.04	0.96	1.13	.241
Stroke	0.259	1.30	1.19	1.41	<.001
Beta Blockers	0.116	1.12	1.06	1.19	<.001
Smoking status					
Never Smoking	Ref				
Smoker	−0.176	0.84	0.79	0.89	<.001
Ex Smoker	−0.143	0.87	0.78	0.96	.008
Vasopressor use at admission	0.016	1.02	0.84	1.23	.250
Type of Admission					
Medical					
Surgical	−0.102	0.90	0.84	0.97	.009
Renal Replacement Therapy (RRT)	0.154	1.17	1.03	1.31	.012

CI = confidence interval, CPR = cardiopulmonary resuscitation, DIC = disseminated intravascular coagulation, MV = mechanical ventilation, NG = nasogastric, PEG = percutaneous endoscopic gastrostomy.

## 4. Discussion

This study is the first to investigate factors associated with prolonged ICU stay in Jordan. Several markers of critical illness severity were strongly associated with prolonged ICU LOS. Patients who required mechanical ventilation, vasopressor support, tracheostomy, renal replacement therapy, or who developed sepsis, septic shock, or disseminated intravascular coagulation (DIC) experienced significantly longer stays in the ICU. In addition, factors such as antibiotic use and enteral access (NG/PEG tubes) were independently determinants of prolonged ICU stay likely reflecting underlying illness complexity. On the other hand, being a smoker or ex-smoker and having surgical admissions were found to be associated with shorter ICU stays.

Mechanical ventilation (or CPAP) at admission was significantly associated with longer estimated average ICU stay in the present study. This is consistent with the findings of a systematic review and meta-analysis that included 28 studies in which patients with MV were found to have higher ICU stays.^[19]^ One possible explanation is that 10% to 20% of patients receiving mechanical ventilation develop ventilator-associated pneumonia (VAP), a complication that significantly prolongs ICU stay. This was observed in a systematic review in which patients with VAP had a mean increase in ICU length of stay of approximately 6.10 days (95% CI, 5.32–6.87 days) leading to an additional ≥$10,019 hospital cost.^[20]^ Similar to our findings, a prospective investigation demonstrated that both mechanical ventilation and vasopressor therapy were associated with nearly 2-fold increases in the odds of prolonged ICU length of stay (mechanical ventilation: OR 1.9, CI 1.3–2.9; vasopressors: OR 1.8, 95% CI 1.2–2.7).^[21]^ The association between vasopressor use and prolonged ICU stay may be partly explained by the increased risk of pressure ulcer development among patients receiving vasopressors.^[22]^

Interestingly, patients who underwent tracheostomy in our study tended to have a substantially longer estimated average ICU stay compared to those who did not. This finding may indicate that tracheostomy has been performed late after intubation, as the literature reported that the timing of tracheostomy significantly impacts ICU LOS, with early tracheostomy (<14 days from intubation) being associated with shorter ICU and hospital stays, whereas late tracheostomy predicts prolonged LOS.^[23]^ Moreover, early tracheostomy placement has been linked to lower reintubation rates, improved discharge to rehabilitation, and lower mortality rates.^[23,24]^ Despite these promising results, it is essential to appropriately select patients for this procedure, because early tracheostomy carries a higher risk of procedural complications, underscoring the importance of individualized, case-by-case decision-making.^[24]^

Receiving antibiotics was significantly associated with a longer ICU LOS in the present study. Several factors can play a role in this association as shown in previous research that has identified antibiotic exposure as a major risk factor for the development of multidrug-resistant organisms in ICUs, which may result in a prolonged ICU stay.^[25]^ Moreover, delayed initiation of appropriate antibiotic therapy has been independently associated with increased post-infection ICU LOS among culture-positive sepsis patients.^[26]^ Inappropriate antibiotic selection could further explain this association, as it often results in prolonged antibiotic therapy, and subsequently, an extended LOS.^[27]^ These findings emphasize the importance of optimizing the timing, duration, and selection of antibiotics in ICU patients to improve their clinical outcomes and reduce LOS.

The current study found that the use of an NG tube for nutrition administration was significantly associated with an estimated prolonged average ICU LOS. Consistent results were reported in earlier research.^[28,29]^ Although NG tubes are commonly used for temporary enteral nutrition in hospitals,^[30]^ they have been associated with serious and potentially fatal adverse events, resulting in poor patient outcomes, increased LOS and even death.^[28,31]^ Furthermore, the need for enteral feeding via NG tubes often reflects more severe underlying conditions, which contributes to extended ICU LOS.^[32]^ Incorrectly placed NG tubes have also been linked to severe complications that can further prolong ICU stay.^[33]^ These findings emphasize the need for effective strategies to ensure correct placement of NG tubes, prevent NG tube–related complications, and thus, reducing ICU LOS. Future research is also warranted to determine whether optimizing the timing and duration of feeding via NG tubes can further decrease ICU LOS and improve patient outcomes.

Consistent with previous research, patients who developed critical complications such as sepsis or organ dysfunction experienced significantly longer ICU stays in our study. In a cohort study from Turkey, patients who developed acute kidney injury (OR = 1807, 95% CI 1434-2277) and pressure sores (OR = 3572, 95% CI: 2663-4792) were significantly more likely to have prolonged ICU stay.^[34]^ Another study among patients with trauma reported that complications such as renal failure, respiratory failure, and sepsis significantly influenced ICU LOS, highlighting the importance of implementing effective preventive strategies in order to prevent complications, reduce the ICU stay, and optimize patients’ outcome.^[35]^

The association observed with current smoking in our study is consistent with the findings of a Turkish study in which the average ICU length of stay was 9.3 ± 8.8 days among nonsmokers and 7.6 ± 5.8 days among current or former smokers, however, the association did not reach statistical significance in their analysis (*P* = .329).^[36]^ These findings were inconsistent with the findings of a retrospective cohort study in which the smoking cohort of consecutive surgical spine trauma patients had a significantly longer ICU length of stay (11.0 ± 12.0 days vs 8.01 ± 7.98 days, *P* = .046) compared to nonsmokers. The association observed in our study should be further investigated as it may be due to unmeasured confounding. Similarly, shorter ICU stay in our study was found to be associated with surgical admissions compared to medical admissions perhaps reflecting the more predictable clinical course and recovery seen in surgical cases.^[37]^

While the present study encompassed ICU admissions from a tertiary hospital over a decade, resulting in a substantial sample size that enhances its generalizability, certain limitations must be acknowledged. Firstly, the reliance on electronic health records limited access to standardized severity of illness scores such as Sequential Organ Failure Assessment score. The absence of these scores may have limited the model’s ability to fully adjust for baseline disease severity. Furthermore, the study primarily focused on identifying predictors of ICU length of stay and did not examine longer-term outcomes such as mortality, post charge functional status, or psychological well-being. Therefore, the broader implications of prolonged ICU stay for patient outcomes beyond the ICU setting remain to be determined. Moreover, while efforts to account for known confounders such as age and comorbidities, the inherent limitations of observational studies must be considered. Notably, variables like socioeconomic factors were not captured in the dataset, which means their potential influence on ICU LOS remains unadjusted. These limitations underscore the need for further research to comprehensively understand the complex interplay of factors influencing ICU LOS and its broader implications for patient outcomes.

## 5. Conclusion

This study identified factors contributing to the LOS in ICUs. Antibiotic use, tracheostomy, NG/PEG tube use, mechanical ventilation, and the presence of complications emerged as significant determinants. Identifying these factors is crucial for optimizing patient management and outcomes, as well as guiding the efficient allocation of healthcare resources. Further research is warranted to examine targeted interventions aimed at reducing LOS among critically ill patients.

## Author contributions

**Conceptualization:** Reema Karasneh, Sayer Al-Azzam, Karem H. Alzoubi, Lana Rababah, Dua’a Mohammad, Dania Rahhal, Louai Alsaloumi, Suad Kabbaha, Mamoon A. Aldeyab, Ohoud Aljuhani.

**Data curation:** Reema Karasneh, Sayer Al-Azzam, Karem H. Alzoubi, Lana Rababah, Dua’a Mohammad, Dania Rahhal, Louai Alsaloumi, Suad Kabbaha, Mamoon A. Aldeyab, Ohoud Aljuhani.

**Formal analysis:** Reema Karasneh, Sayer Al-Azzam, Lana Rababah, Suad Kabbaha.

**Funding acquisition:** Reema Karasneh, Sayer Al-Azzam.

**Investigation:** Reema Karasneh, Sayer Al-Azzam, Karem H. Alzoubi, Lana Rababah, Dua’a Mohammad, Dania Rahhal, Louai Alsaloumi, Suad Kabbaha, Ohoud Aljuhani.

**Methodology:** Reema Karasneh, Lana Rababah, Dania Rahhal.

**Project administration:** Reema Karasneh, Karem H. Alzoubi, Dua’a Mohammad, Suad Kabbaha, Mamoon A. Aldeyab.

**Resources:** Reema Karasneh, Karem H. Alzoubi, Dua’a Mohammad, Louai Alsaloumi, Mamoon A. Aldeyab.

**Software:** Dua’a Mohammad, Dania Rahhal, Louai Alsaloumi, Ohoud Aljuhani.

**Supervision:** Reema Karasneh, Sayer Al-Azzam, Karem H. Alzoubi, Dua’a Mohammad, Louai Alsaloumi, Suad Kabbaha, Mamoon A. Aldeyab, Ohoud Aljuhani.

**Validation:** Reema Karasneh, Sayer Al-Azzam, Karem H. Alzoubi, Lana Rababah, Dua’a Mohammad, Dania Rahhal, Louai Alsaloumi, Suad Kabbaha, Mamoon A. Aldeyab, Ohoud Aljuhani.

**Visualization:** Sayer Al-Azzam, Karem H. Alzoubi, Lana Rababah, Dua’a Mohammad, Dania Rahhal, Louai Alsaloumi, Suad Kabbaha, Mamoon A. Aldeyab, Ohoud Aljuhani.

**Writing – original draft:** Reema Karasneh, Sayer Al-Azzam, Lana Rababah, Dua’a Mohammad, Dania Rahhal, Louai Alsaloumi, Mamoon A. Aldeyab, Ohoud Aljuhani.

**Writing – review & editing:** Reema Karasneh, Karem H. Alzoubi, Suad Kabbaha.
